# Integrating computational fluid dynamic, artificial intelligence techniques, and pore network modeling to predict relative permeability of gas condensate

**DOI:** 10.1038/s41598-022-24468-z

**Published:** 2022-12-12

**Authors:** Ehsan Zeinedini, Bahram Dabir, Mitra Dadvar

**Affiliations:** Chemical Engineering Department, School of Material Engineering and Advanced Processes, Amirkabir University of Technology, Tehran, Iran

**Keywords:** Chemical engineering, Mathematics and computing

## Abstract

The formation of gas condensate near the wellbore affects the gas liquid two-phase flow between the pores. It may occur in the path between two pores depending on the thermodynamic conditions of the single-phase gas flow, two-phase gas liquid annular flow or the closed path of condensate in the throat. To model the behavior of gas condensate in a network of pores, relative permeability and naturally pressure drop should be calculated. This study obtained the flow characteristics (pressure drop) between the pores at different physical and geometric conditions using computational fluid dynamics (CFD). CFD is time-consuming, so its results were transferred to an artificial neural network (ANN) model and the ANN model was trained. The CFD was replaced with the ANN model for calculating the pressure drop. In addition, instead of utilizing empirical correlations to compute the accurate value of condensate formed in throats' corners at every time step, the flash calculation using Esmaeilzadeh–Roshanfekr equation of state was performed, and closed throats were specified. This accurately estimates gas and condensate distribution in the pore network. Furthermore, the value of condensate that transferred to the adjacent throats was computed using Poiseuille's law. The results showed that the proposed ANN-based proxy model could promote the calculation speed in gas condensate simulation, considering the dynamic change of relative permeability curves as a function of gas condensate saturation. Also, it was found that the relative permeability obtained by the proposed model is in good agreement with the experimental data. By entering the fractures pattern in the network model and predicting the relative permeability of gas and condensate by the proposed method, the role of fractures in gas condensate production in such reservoirs could be predicted. Dynamic changes due to the relative permeability of gas and condensate as a function of saturation can be entered into the reservoir simulation to optimize inertia and positive coupling phenomena to maximized condensate production in gas condensate reservoir.

## Introduction

Naturally, gas condensate reservoirs consist of single-phase gas. The liquid phase appears during gas production due to pressure drop below the dew point. As the pressure drop decreases, the rate of liquid phase formation increases. Depending on the amount of condensate formed (liquid to gas ratio), different modes of flow may occur in the pore of porous medium: 1- the two-phase flow of gas–liquid 2- the annular flow of gas–liquid 3- static liquid phase and gas flow from a smaller pore 4- corner liquid-center gas flow 5- obstruction of the path by liquid phase^1^. Depending on the pores geometry, the path between them and the rate of liquid formation, one of the mentioned modes may happen. When the gas condensate formation and liquid phase accumulation around the wellbore is reduced, the effective permeability of gas into the wellhead and consequently the rate of exploitation of these reservoirs increases. Many researchers have tried to understand multiphase flow in such reservoirs to determine the effect of gas condensate accumulation and blockage of voids^2–4^. Various quasi-analytical models have been proposed to analyze the production rate of gas condensate reservoirs^5,6^. In some studies^7,8^, by considering more parameters, it has been tried to correct the relative permeability relationship and improve the accuracy of the models.

In order to investigate the details of flow in porous media, the pore networks model has been used^9^ in which a random pore networks and paths for the porous environment has been considered and different flow phenomena in the paths have been investigated. In most studies of the pore networks model, simple relationships have been used to describe the flow within the porous paths (between the pores). These models have been modified according to the effect of velocity and interfacial tension on relative permeability.

Recently, Momeni et al.^10^ and Reis et al.^11^ used Hagen–Poiseuille equation to calculate the pressure field in the pore network. In these previous works, it was assumed that the flow in the gas condensate reservoir is laminar. Although the application of Hagen–Poiseuille relation in a pore network has good agreement with the experimental results, ignoring the inertial force in sandstone gas condensate reservoir, especially in the near wellbore region where the turbulent flow is high, cannot be ignored and the effect of inertia and convection term is significant. Hence, simplifying the physics of phase transitions and complex fluid composition between gas and condensate is commonly inaccurate. Also, the isothermal flash calculation was used in each step to determine the physical properties. Song et al.^12^ proposed a thermodynamic phase equilibrium model considering phase behavior change at different pressure and temperature. They represented a pore network multiphase multicomponent hydrocarbon transport model developed based on the proposed thermodynamic phase equilibrium calculation model that simultaneously considers the influences of capillary pressure on phase equilibrium and liquid–gas distribution in irregular throat cross-sections. In the previous model performed by Momeni et al.^10^, the Hagen-Poiseuille relation was used to determine the pressure drop and subsequently calculate the relative permeability instead of empirical relations for the prediction of relative permeability. Understanding the concept of multiphase flow in condensate gas reservoirs is a key parameter in condensate dropout and investigating the effect of condensate blockage.

The purpose of this study is to identify the behavior of the gas-condensate system and the parameters affecting the deliverability of these reservoirs. In this research, in addition to using the advantages of Momeni et al.^10^ work compared to past models, using the Navier–Stokes equations, the effects of capillary, inertia and viscosity parameters on the flow between the paths on the calculation of pressure drop and two phase gas condensate flow mechanism are explored. The flow characteristics (pressure drop) between the pores at different physical and geometric conditions were obtained using computational fluid dynamic. CFD is time-consuming, its results are transferred to an artificial neural network (ANN) model and the ANN model is trained. The CFD is replaced with the ANN model for calculating the pressure drop. In addition, instead of utilizing empirical correlations to compute the accurate value of condensate formed in throats' corners at every time step, the flash calculation using Esmaeilzadeh–Roshanfekr equation of state is performed. Using flash calculation, closed throats are specified. This accurately estimates gas and condensate distribution in the pore network. In addition, the value of condensate that is transferred to the adjacent throats is computed using Poiseuille's law. This algorithm calculates the reservoir's absolute and relative permeability versus different condensate ratios to gas It can also be applied to different types of porous media and fluids. It does not need any experimental study to determine the empirical information about gas and condensate flow behavior.

## Modeling steps

**Figure 1:**
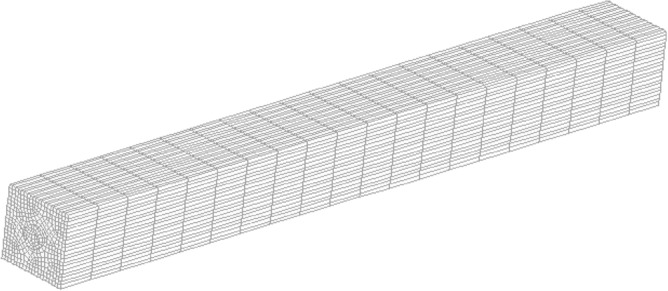
Three-dimensional grid meshed using unstructured hexahedral elements.

The aim of this study is as follows. In the first step, to solve the Navier–Stokes equations numerically, a three-dimensional grid has meshed with unstructured hexahedral elements (Fig. 1).

The various dimensions of the throat cross-section were selected to cover the entire range of the throats radius (R) by the Weibull distribution. Then, using computational fluid dynamic (CFD), the flow characteristics (pressure drop) between the pores at different physical and geometric conditions were obtained. The results obtained from CFD were transferred to an ANN model (as the proxy model) in two parts of training and testing. The ANN model was used for calculating the pressure drop instead of CFD. This significantly reduces computational costs.

Then, a random pore network was created. The flash calculation was employed in each step to specify the amount of fluids in every throat. The physical properties of the phases in a throat were calculated based on the composition and amount of phases. Subsequently, the results obtained were transferred to the ANN to determine the pressure drop of each of the paths between the two pores. This process was repeated between all network pores to obtain the overall network pressure drop based on the inlet gas and condensate flow rate. This algorithm was used to calculate the absolute and relative permeability of the reservoir versus different ratios of condensate to gas. In the next section, the details of the modeling processes are presented.

### CFD modeling

The mechanism of multiphase flows at different operating conditions of gas-condensate reservoirs is a key parameter in determining well deliverability. Thus, the effect of all physical parameters of the subject must be investigated. So far, the pressure drop between the throats has been calculated using empirical correlations or a simple equation such as Hagen-Poiseuille. In this study, accurate calculations of flow properties in a porous medium were solved by considering the direct effect of physical quantities such as interfacial tension, viscosity and density in the form of 3D Navier–Stokes equations in the path between two pores.

The continuity and momentum equations of gas and condensate flow based on the one-fluid method are expressed as follows, respectively:

1 $$\nabla .u = 0$$

2 $$\rho \frac{{Du_{x} }}{Dt} = - \nabla P - \nabla .\tau + f$$

where $u$ is the flow velocity, ρ is the flow density, $P$ is the pressure and $\tau$ is the viscous stress tensor.

The $f$ term represents all external forces, i.e., gravity and capillary forces, defined by the following relations, respectively:

3 $$f_{g} = \rho g$$

4 $$f_{c} = \delta \left( n \right)\sigma \left( {\nabla .n} \right)n$$

In this investigation, the term of gravity in the equations is ignored due to the small size of the studied throats (length less than one millimeter).

The boundary conditions of the velocity at the throat inlet in the y and z directions are zero and a constant value is considered in the x direction:

5 $$\begin{aligned} ub(in) & = Fixed \\ vb(in) & = 0 \\ wb(in) & = 0 \\ \end{aligned}$$

Besides, at the throat outlet, the same pressure is regarded for the two phases:

6 $$pex(out) = Fixed$$

Fixed pressure is the defined pressure to obtain only the pressure drop of a throat, which should have been defined to calculate the pressure drop in a throat. In addition, no slip boundary condition is considered for the walls.

Considering the convection term (inertia term) typically causes the equations to be non-linear. It is not easy to solve these equations. For this reason, in all previous works^6,10,11,13^, this term has been omitted and it is assumed that the gas condensate reservoir flow is laminar flow. Therefore, in previous studies^10,11^, the behavior of gas and condensate flow in the throats has been investigated using Hagen-Poiseuille equation. Ignoring the inertial force is expected in the oil reservoir. However, in the sandstone gas condensate reservoir, especially in near a wellbore region where the turbulent flow is high, the effect of convection term is significant.

In order to solve these equations, the finite volume discretization method was used^14–16^. In this study, a pressure-based finite-volume scheme for unstructured meshes was developed. The combined use of a finite-volume discretization with a segregated SIMPLE algorithm was employed and used for velocity–pressure coupling via a pressure correction equation. The SIMPLE (Semi-Implicit Method for Pressure-Linked Equations) algorithm is fundamentally a guess-and-correct method for computing pressure on the non-staggered grid. The concept of the SIMPLE algorithm is to create a discrete equation for pressure from the discrete continuity equation^17,18^.

Finally, the density and viscosity of fluid were defined as follows for the flow of gas and condensate using the characteristic function:

7 $$\begin{gathered} \rho = \alpha \rho_{1} + \left( {1 - \alpha } \right)\rho_{2} \hfill \\ \mu = \alpha \mu_{1} + \left( {1 - \alpha } \right)\mu_{2} \hfill \\ \end{gathered}$$

where $\alpha$ is indicator function. The indicator function ($\alpha$) shows the volume fraction of one of the phases in each grid. If the grid is occupied with the gas phase then $\alpha$ = 1 and if it is occupied with the condensate phase $\alpha$ = 0. For grids containing the interface bounding phases, $\alpha$ lies between zero and unity.

In order to solve the Navier–Stokes equations of multiphase flow and obtain the characteristic function, the major problem is the tracking of the interphase surface between fluids in multiphase flow. In this research, to solve this problem, a couple the fluid volume (VOF) and level set methods have been used for interfacial flow simulations^19,20^, which takes advantage of both VOF and level set methods.

At each step, the level set function and the VOF are evolved by computing the level set advection equation and the VOF advection. The interface was reconstructed according to both the level set and the VOF data. Actually, the line constant was obtained using enforcing mass conservation from the VOF, while interface normal vector was determined based on the level set function.

The term of capillary force as volumetric force should be calculated as follows:

8 $$f_{c} = \sigma \kappa n_{s} \delta_{s}$$

where $\kappa = \nabla .\left( {n_{s} } \right)$ is the curvature of the common interphase. In addition,$n_{s}$ is the normal vector on the interface defined as:

9 $$n_{s} = \frac{\nabla \alpha }{{\left| {\nabla \alpha } \right|}}$$

The $\alpha$ term is a characteristic function. As described above, the normal interface vector was calculated using the continuous gradient of the level set function. For computing, the normal vector, a quadratic form of $\alpha$ based on least squares approximation including of the vertices of the cell and the neighboring cells that have a common vertex with the cell has been applied^21^.

Finally, $\delta_{s}$ is the delta-focused function of the interface, which is defined as follows:

10 $$\delta_{\varepsilon } \left( \phi \right) = \frac{{dH_{\varepsilon } }}{d\phi }$$

The Heaviside function $H_{\varepsilon }$ is also stated by the following relations:

11 $$H_{\varepsilon } \left( \phi \right) = \left\{ \begin{gathered} 0\quad \quad \quad \quad \quad \quad \quad \quad \quad \quad \quad\;\;if\phi < - \varepsilon \hfill \\ \frac{1}{2}\left[ {1 + \frac{\phi }{\varepsilon } + \frac{1}{\pi }\sin \left( {{\raise0.7ex\hbox{${\pi \phi }$} \!\mathord{\left/ {\vphantom {{\pi \phi } \varepsilon }}\right.\kern-\nulldelimiterspace} \!\lower0.7ex\hbox{$\varepsilon $}}} \right)} \right]\;\;\;if\left| \phi \right| \le \varepsilon \hfill \\ 1\quad \quad \quad \quad \quad \quad \quad \quad \quad \quad \quad \;\;if\phi > \varepsilon \hfill \\ \end{gathered} \right.$$

The thickness of the interface is equaled $2\varepsilon$ according to^21^.

The curvature of the interface is calculated as the divergence of the unit normal vector over the interface:

12 $$\kappa = - \nabla .\left( {\frac{\nabla \alpha }{{\left| {\nabla \alpha } \right|}}} \right) = - \nabla .n$$

The solving method of the equations in this research is based on the work done in literature, the details of which can be found elsewhere^22–24^.

**Figure 2:**
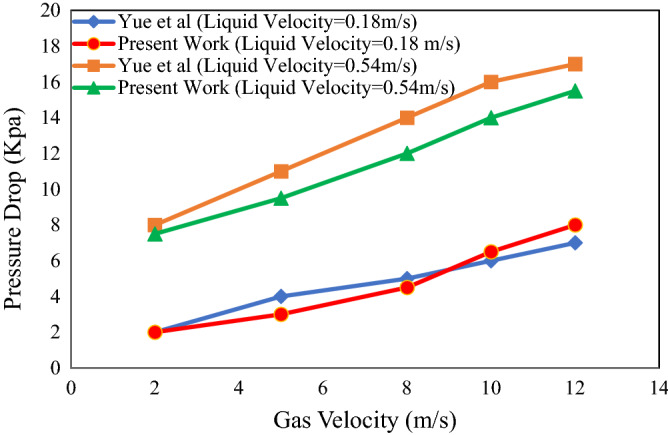
Pressure drop of gas liquid two phase flow versus velocity.

The results of two-phase pressure drop calculations of the model presented in this study were compared with the experimental results performed by Yue et al.^25^ in micro channels. Yue’s et al.^25^ paper calculated the pressure drop of two gas and liquid phases (nitrogen and water). The physical properties of nitrogen–water two-phase flow (including density and viscosity) and geometry (length and diameter) were implemented in the CFD code. The results are presented in Fig. 2. It should be noted that, the model and experimental results showed good quantitative agreement for pressure drop.

Input parameters are length and hydraulic diameter of rectangular microchannel equal to 3.43 cm and 500 μm, respectively. Also, physical properties of nitrogen–water two-phase flow including density and viscosity are considered.

Although CFD provides more reliable results with more details, it is not computationally possible to apply the obtained CFD results to all paths between pores in the network. To solve this problem, artificial neural network capabilities were used to increase the speed of computer calculations to transfer CFD results over a pore network to create a CFD coupling over a network of pores. Considering the hydrodynamic details and physical properties of the phases, the effect of velocity and surface tension is specified on permeability in the area near the wellbore in gas condensate reservoirs. In the computational nodes near the well, with the production from the well, due to changes of the condensate saturation in the formation, these changes can be dynamically used into the calculations of relative permeability curves.

### Transfer of CFD data to the neural network

Direct use of CFD to predict mechanism flow in all pore network paths is not computationally possible. The neural network provides a suitable basis for transferring CFD results to the pore network^26^. For this purpose, Navier–Stokes two-phase equations in different radius, velocities and physical properties were collected for network training.

The selection of the data set for training was selected to covers the entire range of the radius surrounding the throats (R) by the Weibull distribution. It should be mentioned that in all calculations performed, the length of the throats was assumed to be the same. Although the proposed model was applied for constant throat lengths, one may apply the proper length distribution function to determine throat length. Nevertheless, compared to previous works^10,11,13^, the same length was considered in this study.

The process was performed according to the results obtained from the repeating of the different neurons using the values of mean squared errors (MSEs), mean relative errors (MREs) and correlation coefficient of the network outputs ($R^{2}$) as a performance of determining the optimal number of neurons with the best bias and weight from the target function^27,28^. The MSE, MRE and $R^{2}$ relations are defined using the following forms:

13 $$MSE = \frac{1}{N}\sum\limits_{i = 1}^{N} {\left( {\alpha^{calc} - \alpha^{pred} } \right)^{2} }$$

14 $$MRE = \frac{1}{N}\sum\limits_{i = 1}^{N} {\frac{{\left| {\alpha^{calc} - \alpha^{pred} } \right|}}{{\alpha^{calc} }}}$$

15 $$R^{2} = \frac{{\sum\limits_{i = 1}^{N} {\left( {\alpha^{calc} - \overline{\alpha } } \right)^{2} } - \sum\limits_{i = 1}^{N} {\left( {\alpha^{calc} - \alpha^{pred} } \right)^{2} } }}{{\sum\limits_{i = 1}^{N} {\left( {\alpha^{calc} - \overline{\alpha } } \right)^{2} } }}$$

In these relations N, $\overline{\alpha }$, $\alpha^{calc}$ and $\alpha^{pred}$ are the total number of data points, average pressure drop, pressure drop calculated, pressure drop predicted of gas and condensate in the throats, respectively.

**Figure 3:**
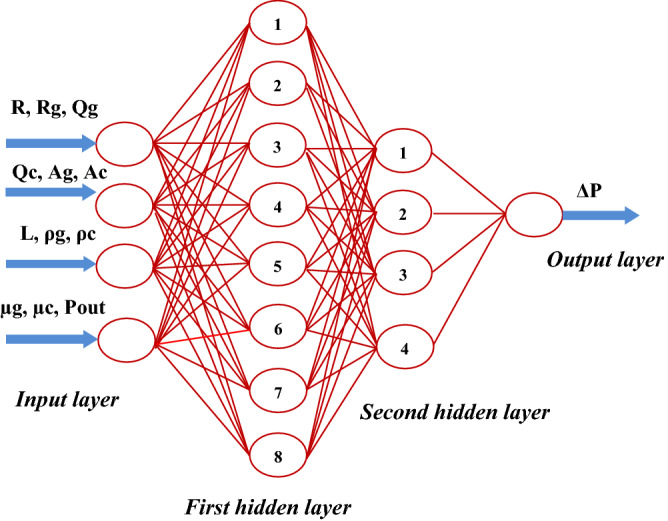
Input data and hidden layers of neural networks.

The database consists 504 data sets of CFD simulations extracted under different conditions. The results are randomly used in two parts of training (75% of the database) and testing (25% of the database) in the neural network. Effective Parameters on the pressure drop in two phase flow are the throat length, radius of each throat, amount of the gas phase and the condensate in throats, flow rate or velocity of the gas phase and the condensate, the physical properties of phases (density and viscosity) and output pressure (Fig. 3). In fact, the mentioned parameters are the specified initial and boundary conditions in the solving of the Navier–Stokes equations. These parameters were also used in the neural network method (according to Eq. 16) to calculate the pressure drop of each throat.

16 $$\Delta P = f\left( {R,R_{g} ,Q_{g} ,Q_{c} ,A_{g} ,A_{c} ,L,\rho_{g} ,\rho_{c} ,\mu_{g} ,\mu_{c} ,P_{out} } \right)$$

where $\Delta P$ and $P_{out}$ are pressure drop and outlet pressure of each throat, respectively. $R$ and $R_{g}$ are the radius of throats and radius of gas at throats, $Q_{g}$ and $Q_{c}$ are inlet volumetric flow rates of gas and condensate to the network. $A_{g}$ and $A_{c}$ are the cross-sectional of gas and condensate, $L$ is the network's length in the direction of flow. $\rho_{g} ,\rho_{c} ,\mu_{g} ,\mu_{c}$ are density and viscosity of gas and condensate in throats. As mentioned, the variation of throat shape is not considered in this study and the square cross-section of the throats was assumed to be the same.

Among the learning algorithms, the combined quasi-second-order method and the Levenberg–Marquardt algorithm was used as a training technique^29^.

The results are obtained by repeating the number of different neurons using the values of MSE, MRE, R^2^ as basis for determining the number of optimal neurons. The best network structure with the least error and the best R^2^ were selected. The optimal values of MRE, MSE, R^2^ and epoch for training and test data sets for gas and condensate pressure drop are reported in Table 1.

**Table1:** The optimal values of MRE, MSE, R2 and epoch for training and test data sets.

Pressure drop	Train data set				Test data set		
	MRE	MSE	R^2^	Epoch	MRE	MSE	R^2^
Gas Phase	1.2092	1.9761e−05	1.0000	325	2.6892	2.4512e−05	0.9997
Condensate Phase	3.5190	2.1599e−04	0.9957	1157	5.7717	5.1599e−04	0.9858

The network with two hidden layers consists of 12 neurons for pressure drop of gas and one hidden layer including 12 neurons for pressure drop of condensate, had very good performance.

**Figure 4:**
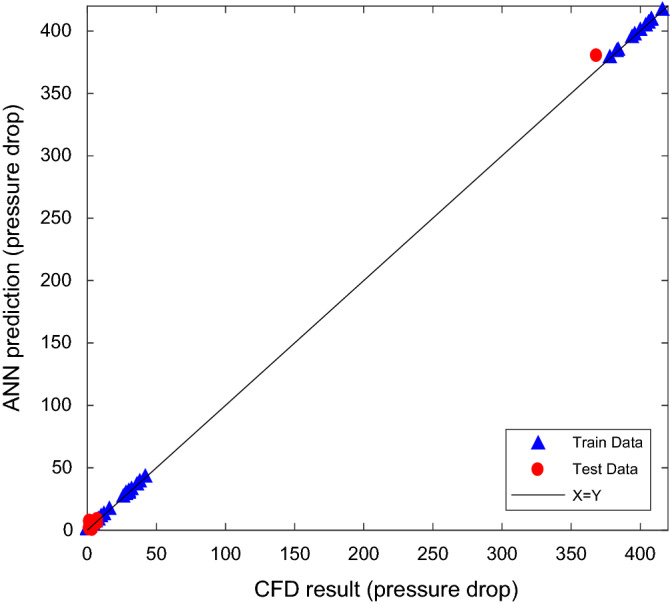
Validation of predicted versus calculated gas pressure drop for train and test sets.

**Figure 5:**
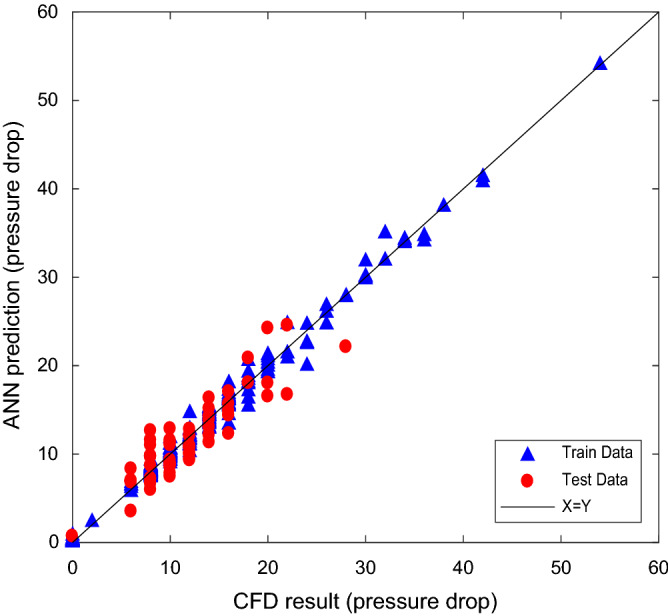
Validation of predicted versus calculated condensate pressure drop for train and test sets.

To evaluate the accuracy of ANN, the Navier–Stokes two-phase equations and ANN predicted data were compared for all throats in graphs shown in Figs. 4 and 5. In both figures, x demonstrates the calculated pressure drop of gas and condensate in the throats based on CFD calculation and y demonstrates the predicted values by ANN. The diagonal line is the geometric location of the coordinate with the least percentage error.

Deviation from the diagonal line indicates the difference between the output of the neural network and the data obtained from the CFD at the same input conditions. 126 data not applied in the training phase were used to test the performance of the developed network.

According to the range of throat sizes from 1.3 to 19.2 microns based on throat size distribution, most throat sizes are from 3 microns and above. In addition to the wide range of throat sizes, the high number of effective parameters on the pressure drop calculation is caused to increase computational costs. Therefore, to reduce the computational time and be more accurate in the middle area of the throat size, more pressure drop calculations have been performed in this area. Therefore, it caused not to have the pressure drop in the middle area in Fig. 4. If the number of calculations were increased for smaller throat sizes, the pressure drop would be between 50 and 350 kpa.

### Phase equilibrium in throats based on the Esmaeilzadeh and Roshanfekr (ER) thermodynamic equation

After calculating the pressure drop obtained by ANN, to find out the formation of the condensate in different throats, using this pressure and the calculations of flash calculation, the presence of condensate in each throat was detected. Then, the amount of each phase and their composition in each throat was obtained. Therefore, if there is condensate in a throat, the two-phase equations of the gas and liquid were solved.

The effects of the molecular weight of hydrocarbons and the mass transfer between the gas and liquid phases were applied through the thermodynamic equation. While the effect of velocity on the relative permeability, which includes the positive coupling effect, was applied through Navier–Stokes equations. Considering the Navier–Stokes equations as compositional requires very high computational time and advanced computer facilities.

In this study, the ER-EOS three-parameter cubic equation of state was used instead of the Peng Robinson (PR) EOS for the thermodynamic modeling of reservoir fluids. The predictions of this equation for the PVT properties of light and intermediate hydrocarbons are better than other equations^30,31^. The ER-EOS has the following form:

17 $$P = \frac{RT}{{v - b}} - \frac{a\left( T \right)}{{v\left( {v + c} \right) + c\left( {v - c} \right)}}$$

where R is the universal gas constant, “a” is a function of temperature and “b” and “c” are constants.

The ER-EOS equation results show that saturated liquid and vapor densities are more accurate than the PR-EOS equation for pure components. As well as, the predictions of the saturated vapor pressure of light hydrocarbon components demonstrated that ER-EOS has less error than PR-EOS^32^.

Some assumptions were considered; the temperature of reservoir is constant; the pressure is changed regarding to the fluid flow and the changes in the composition of each phase is possible. It should be noted that gas and condensate in this equation were considered as the compositional and the physical properties of each phase. These parameters were determined based on the composition of that phase.

### Pore network structure and modeling

**Figure 6:**
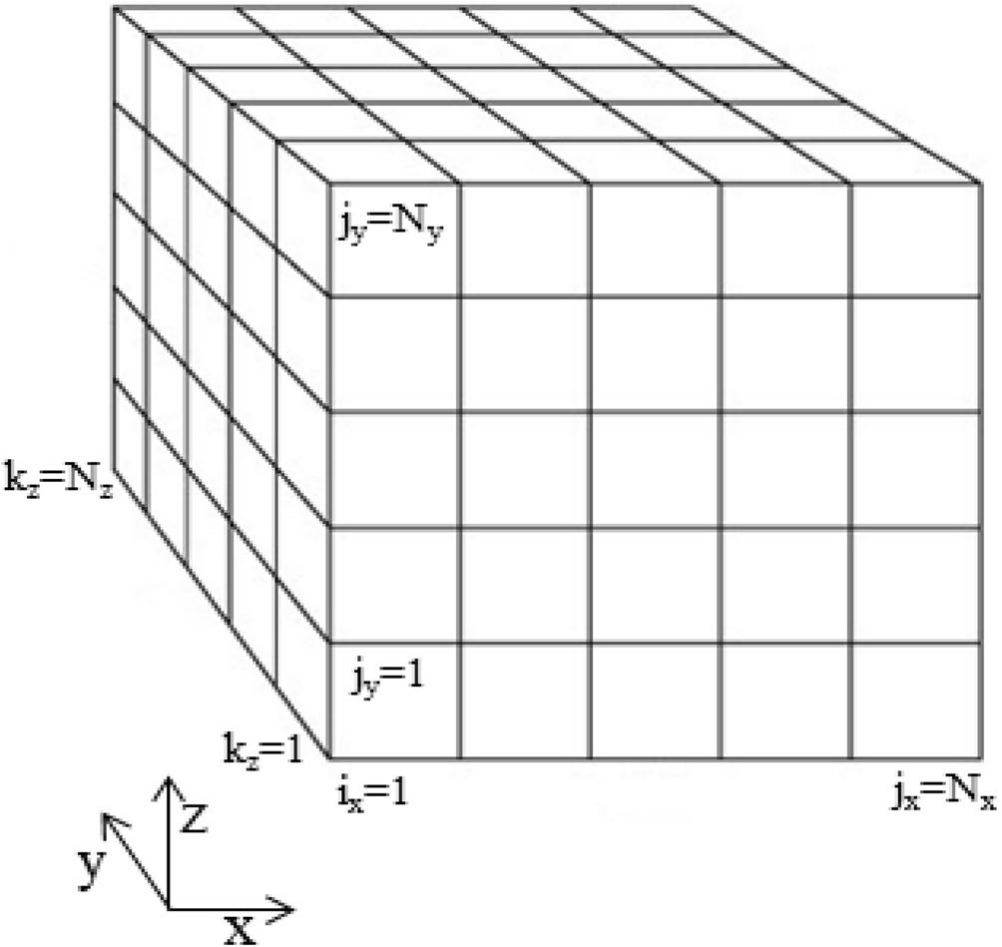
Regular three-dimensional grid consisting of necks with regular polygonal cross section.

In a pore network, the porous medium consists of a network of empty spaces called pore throats (throats), which are connected to each other by the pore bodies (pores). Figure 6 shows an example of a 3D cubic network with square cross sections in which fluids enter the network at the inlet level and exit at the outlet level. For simplification, the volume of the pores was ignored in the calculations.

**Figure 7:**
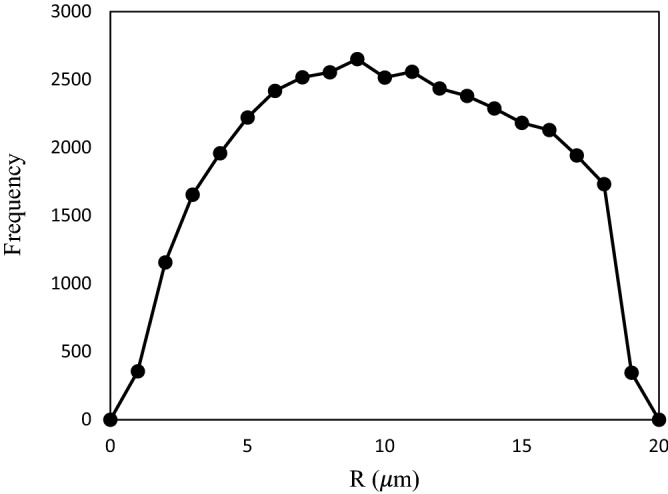
Throat size distribution.

To validate the algorithm by the experimental samples of Jamiolahmady et al.^13^, a three-dimensional pore network of square-shaped capillary tubes was considered with dimensions of 25 × 25 × 20 and absolute permeability of 92 mD. In the whole network, the length of the throats is the same and equal to an average value. Of course, if sufficient information is available about the structure of the porous medium, the length of the throat can also be determined from a suitable distribution function. The Weibull distribution function^33^ was used to determine the radius of the throat randomly as follows:

18 $$R = \left( {R_{\max } - R_{\min } } \right)\left( { - \delta \ln \left[ {x\left( {1 - e^{{ - {1 \mathord{\left/ {\vphantom {1 \delta }} \right. \kern-\nulldelimiterspace} \delta }}} } \right) + e^{{ - {1 \mathord{\left/ {\vphantom {1 \delta }} \right. \kern-\nulldelimiterspace} \delta }}} } \right]} \right)^{{{1 \mathord{\left/ {\vphantom {1 \gamma }} \right. \kern-\nulldelimiterspace} \gamma }}} + R_{\min }$$

where R is the radius, $R_{\max }$ and $R_{\min }$ are the maximum and minimum radius, respectively. In addition, $\delta$ and $\gamma$ are the parameters of distribution defined for any kind of core. Besides, x is a random number. These parameters are presented in Table 2 and Fig. 7 shows throat size distribution.

**Table 2:** Weibull distribution parameters for throat^33^.

parameter	Throat
$\delta$	0.8
$\gamma$	1.6
$R_{\max }$	1.3
$R_{\min }$	19.2

The cross-sectional area of regular polygons is defined as follows^33^:

19 $$A = \frac{{NR^{2} }}{\tan \alpha }$$

where N and $\alpha$ are the number of throats' corners and a corner’s half angle, respectively. In this study, N is considered to be four. Also, $\alpha$ is determined as follows:

20 $$\alpha = \frac{\pi }{2}\left( {\frac{N - 2}{N}} \right)$$

A mixture of 75% for methane and 25% for normal butane was regarded similar to Jamiolahmady et al.^13^. Other physical characteristics are listed in Table 3.

**Table 3:** Surface tension and viscosities of gas and condensate at the used pressure in the network (at 37 °C) Jamiolahmady et al.^13^.

Pressure $\left( {MPa} \right)$	viscosities of condensate $\left( {{\text{mpa}}\;{\text{s}}} \right)$	viscosities of gas $\left( {{\text{mpa}}\;{\text{s}}} \right)$	Surface tension $\left( {{\text{mN}}\;{\text{m}}^{ - 1} } \right)$
13.04	0.0351	0.0226	0.015
12.78	0.0371	0.0214	0.037

The boundary conditions at the inlet and outlet of pore network are defined as follows:

21 $$\begin{aligned} & Q_{gas - in} = Fixed \\ & CGR_{in} = Fixed \\ & P_{gas - out} = P_{con - out} = Fixed \\ \end{aligned}$$

In y and z boundaries, periodic boundary conditions are considered.

### Conditions of open and closed throats

At each step, the closing and opening of the throats is computed based on the equations of pressures $P_{C}$, $P_{Sth}$ and $P_{entry}$ for a cubic throat. When the pressure alters between the gas and condensate in an inlet node to the throat and it is greater than the capillary pressure in that throat (Eq. 22), the throat is opened. After opening the throat, the existing condensate is transferred to the adjacent throats. The amount and ratio of condensate transfer to each of the throats neighboring according to the CFD results through the ANN was determined based on the pressure difference, the radius of the throats, and the amount of condensate in the neighboring throats. The $P_{entry}$ is expressed by the following relation:

22 $$P_{entry} = P_{C} - (P_{g} - P_{l} )$$

The local capillary pressure is defined as follows:

23 $$P_{C} = \frac{\sigma }{{R_{w} }}$$

where $R_{w}$ is the radius of curvature of the wetting film at the corners.

When a number of the throats are closed, the amount of condensate transferred by the opening of the throat to each of the surrounding throat can be estimated by flash calculation. In open throats, as the amount of condensate increases, the local capillary pressure in that throat gradually decreases. This process continues until the local capillary pressure is less than the snap-off pressure phenomenon of a square cross section throat (Eq. 24). Increasing the amount of condensate in a throat eventually causes the throat to be closed^34^. The following equation is the snap-off pressure threshold:

24 $$P_{Sth} = \frac{\sigma }{2R}\left( {\cos \theta - 2\sin \theta } \right)$$

Sometimes in the network, the throats may be closed so that the gas flow through the entire network might be eliminated. In the event of this phenomenon, the closed throats by condensate, which are in contact with the gas entering the network, will open continuously (beginning of the throat that has the lowest $P_{entry}$ according to the following equation^13^:

25 $$P_{entry} = P_{c} + P_{l}$$

This process continues until there is a suitable path for gas movement in the network^13^.

### Simulation

After forming a pore network and determining the input conditions, at the starting of the simulations, the outlet pressure of the pore network was considered above the dew point. Hence, there was only the gas phase in the network. The absolute permeability of the pore network was computed as 92 mD. As the outlet pressure of the pore network is lowered, the gas pressure degrades below the dew point and condensate phase is formed in pore network.

Then, gas and condensate were injected at the inlet of the pore network at fixed flow rate and the specified outlet pressure. The amount of fluids for each throat was obtained based on the flash calculation.

Subsequently, new distributions of fluids in the network were identified and the relative permeabilities of gas and condensate were computed. This condition was used for subsequent time intervals^10^. Then, step by step, the flow mechanism configuration in network was found and the gas and condensate pressure drop, and as a result, the relative permeabilities of gas and condensate were updated.

As for the network size of 25 × 25 × 20, it includes 38,000 throats and 12,500 pores. Thus, at different time steps, 38,000 times thermodynamic equation was used for throats and two sets of pressure equations $n_{x} \times n_{y} \times n_{z} + 1$ were formed for the pressure drop of gas and condensate which lead to the formation of a sparse linear system. The algorithm for solving the equations was the restarted Generalized Minimum Residual (GMRES) algorithm. The pressure drop matrix constants are specific values determined by the node's position relative to neighboring nodes and unknown values are the pressure of each node. Therefore, the values of pressure in each node and finally the inlet pressure can be calculated.

### Calculation of the relative permeability of phases in the computational network

Using ANN and thermodynamic equations in the pores network leads to the calculation of the total pressure drop at a certain flow rate and the total penetration coefficient can be calculated by the following equation:

26 $$k_{rg} = \frac{{\mu_{g} Q_{gas} }}{{Ak_{a} }}\left( { - \frac{L}{{(\Delta P)_{gas} }}} \right)$$

27 $$k_{rc} = \frac{{\mu_{c} Q_{con} }}{{Ak_{a} }}\left( { - \frac{L}{{(\Delta P)_{con} }}} \right)$$

where $K_{rg}$ and $K_{rc}$ are the relative permeabilities of gas and condensate and $K_{a}$ is absolute permeability, respectively. $\Delta P_{gas}$ and $\Delta P_{con}$ are the total pressure drop of gas and condensate and $A$ is the cross-sectional area of the network.

**Figure 8:**
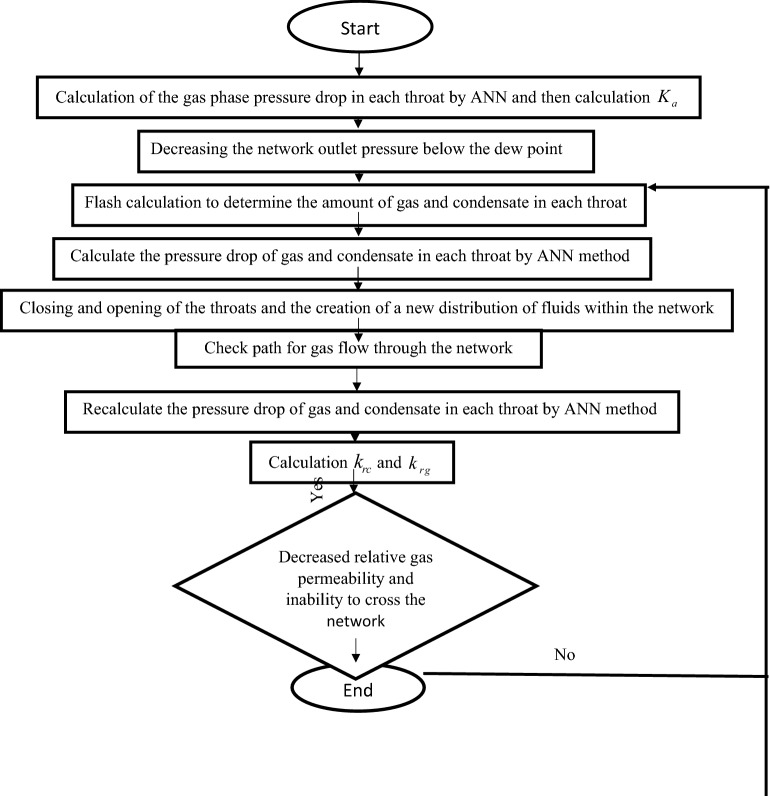
Gas-condensate dynamic flow modeling algorithm.

The algorithm of calculating relative permeability of gas condensate reservoirs is displayed in Fig. 8.

## Results and discussion

**Figure 9:**
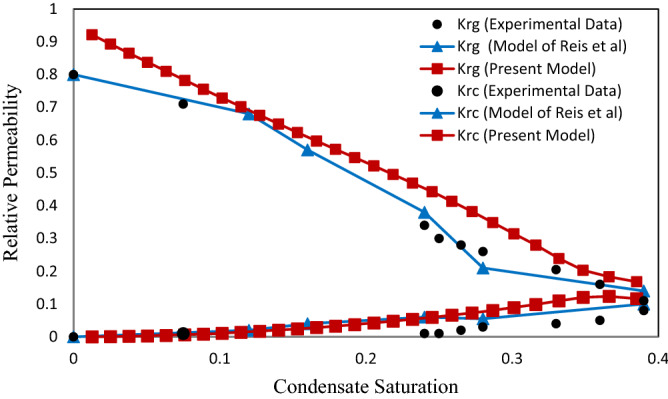
Relative permeability versus saturation at velocity of 18 m/day and IFT = 0.037 mN/m.

**Figure 10:**
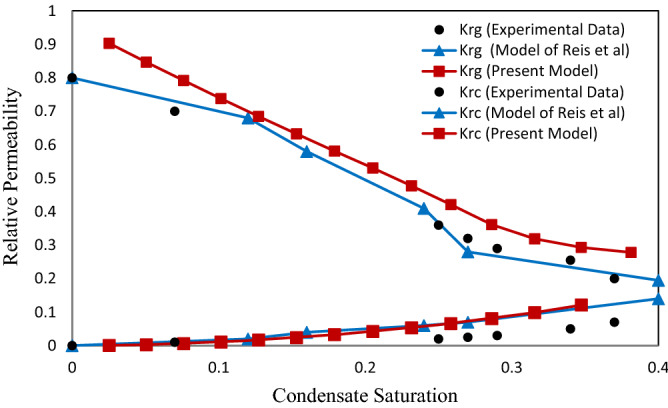
Relative permeability versus saturation at velocity of 36 m/day and IFT = 0.037 mN/m.

**Figure 11:**
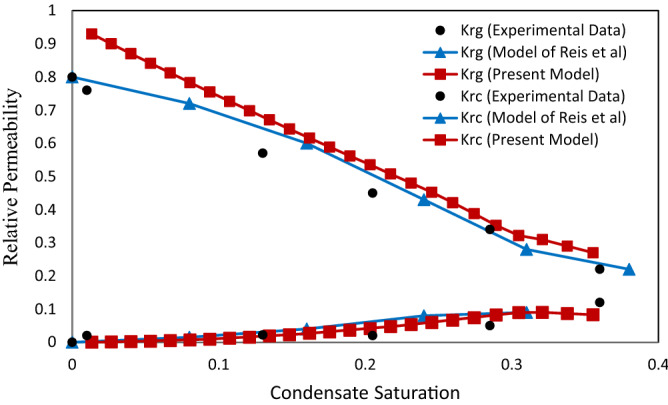
Relative permeability versus saturation at velocity of 18 m/day and IFT = 0.015 mN/m.

**Figure 12:**
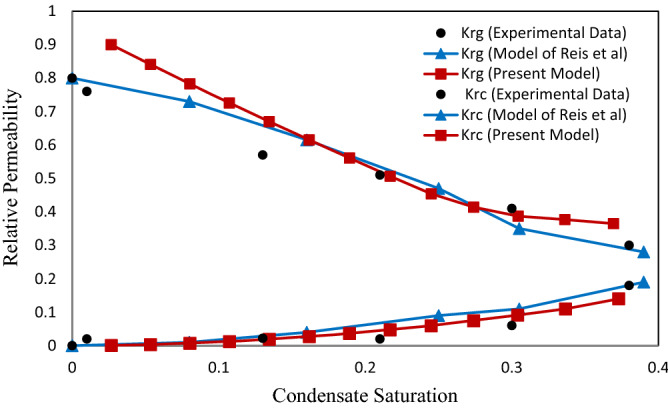
Relative permeability versus saturation at velocity of 36 m/day and IFT = 0.015 mN/m.

As already stated, an ANN model was proposed as an alternative for CFD. To validate the proposed ANN model and pore network, the experimental results carried out by Jamiolahmady et al.^13^ and the data obtained from the Hagen-Poiseuille model^10,11^ were used. A comparison of the effects of velocity and surface tension on the relative permeability of gas and condensate are presented in Figs. 9, 10, 11 and 12. As seen in these figures, the proposed model in this study is in good agreement with the experimental data.

### The effect of gas velocity on the relative permeability

In this section, the gas and condensate relative permeability curves at different inlet flow rates at surface tension of 0.037 mN/m and ratio of condensate to gas flow rates (CGR) of 0.1 were investigated. According to the last investigation of Jamiolahmady et al.^13^ and Reis et al.^11^, the effect of gas velocity on the relative permeability has been investigated at velocities 9, 18 and 36 m/day. Therefore, only the effect of high values of these velocities has been investigated to compare the presented model with them. In the experimental work performed by Jamiolahmady et al.^13^ as well as the model of Reis et al.^11^, due to the presence of irreducible water saturation of 26.4%, (Figs. 9 and 10) the initial relative permeability value has been started from 0.8. While in the present study, there is only gas and condensate phase in the network. Usually, in real conditions there is irreducible water saturation in the formation. Therefore, the experimental results presented by Jamiolahmady et al.^13^ are closer to reality.

The gas relative permeability in the proposed model, especially for saturation values less than 0.25, shows higher values (Figs. 9 and 10). This behavior can be induced by the difference in the geometry of the throat and the porosity of the considered network. Actually, the flow behavior is mainly depended on the porous medium. If sufficient data of porous structure by Jamiolahmady et al.^13^ were available, obtained relative permeabilities of present model are closer to experimental results.

With increasing the saturation, as the flow of condensate inside the throat is annular, condensate grows in the corners, which increases the condensate saturation of the network and as a result the gas pressure drop is increased. The increase of condensate in corners is continued until the throat is wholly saturated with condensate. In other words, it closes the throat (Snap-off). Hence the graph decreases with a sharper slope (Figs. 9 and 10).

As expected, gas and condensate relative permeability in Fig. 10 compared with Fig. 9 increases when the inlet flow rate into the network increases as the positive effect.

In fact, by increasing the gas pressure behind the closed throat, the condensate is pushed out to neighboring throats. Hence the number of closed throats is reduced. Consequently, the total pressure drop in the network decreases and improves relative permeability. This is because viscous forces become more substantial compared to capillary forces. However, the flow rate has less influence on condensate relative permeability.

### The effect of surface tension on the relative permeability

The effect of surface tension on the relative permeability in two values of surface tension 0.037 (mN/m) (Figs. 9 and 10) and 0.015 (mN/m) (Figs. 11 and 12) was investigated. The purpose of presenting Figs. 11 and 12 are compared with the previous experimental data by Jamiolahmady et al.^13^ and the model of Reis et al.^11^, which were illustrated in the same way. Therefore, according to the only available experimental data, investigations have been made on the defined velocities and surface tensions.

As surface tension in Figs. 11 and 12 compared with Figs. 9 and 10 decreases, the effect of velocity on relative permeability increases. In other words, increasing the surface tension increases the capillary force (or decreases the capillary number). This reduces the ability of the gas to reopen the throat, which in turn increases the number of closed throats in the network. Increasing the number of closed throats in the network further increases the pressure drop in the gas phase and consequently reduces the relative gas permeability. This effect is also seen in Figs. 9, 10, 11 and 12.

As observed in Figs. 11 and 12, condensate relative permeability curves obtained in the present work were compared to the curves illustrated based on Reis et al.^11^ investigations. A good quantitative agreement was obtained for the present work results with Jamiolahmady et al.^13^. This can be due to consideration of square cross section for throats that increase of condensate in the throats' corners is resulted.

### Relative permeability of different areas of gas condensate reservoir

**Figure 13:**
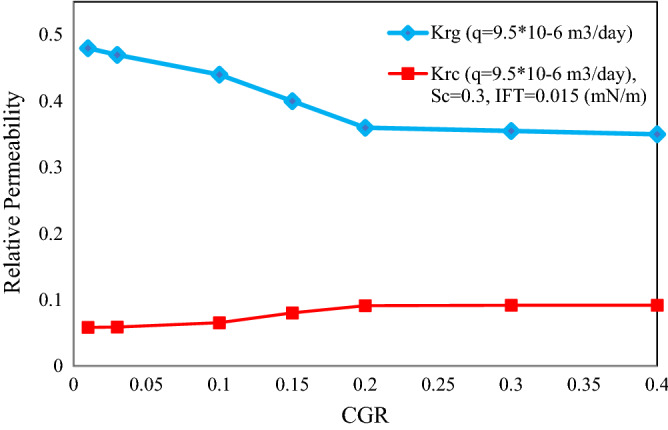
Changes in relative permeability of gas and condensate versus changes in CGR.

Ratio of condensate to gas flow rates (CGR) is an appropriate good criterion for different areas of gas condensate reservoir. Therefore, the effect of CGR ratio on the input flow rate to the pore network on the relative permeability was investigated. Figure 13 shows the variations of gas and condensate relative permeabilities versus the CGR. As expected, with enhancing the condensate flow into the network, the gas permeability decreases and the condensate permeability increases. With increasing CGR, the portion of gas phase in the network decreases and most of the throats are filled with condensate. Thus, the pressure drop is increased in the gas phase and subsequently the relative gas permeability is degraded. For CGR greater than 0.2, the effect of the condensate flow increasing is negligible. The reason of this happening can be related to the ability of the fluid motion decreases for higher values. Hence, the relative permeability is not significantly changed.

## Summary and conclusions

In this study, a three-dimensional pore network model was used to model the dynamic of gas condensate flow near the wellbore region, which applies the effect of high velocities around the well. The presented modeling can be used to investigate the complex gas condensate composition in different porous media. First, the two-phase flow in each throat was solved by the Navier–Stokes equations. The finite volume method was utilized to solve these equations. The velocity and pressure changes for each pore were obtained by solving the Navier–Stokes equation in each throat. In order to reduce the computational time, the output results of $CFD$ models were used in network models using a proxy model (artificial neural network method) that predicts system behavior. Then, flash calculations were performed to accurately calculate the amount of fluids in each throat of the network. Finally, the relative permeability of gas and condensate at each time interval was computed using the Darcy equation. The results at different inlet flow rates and different surface tensions were presented. For two values of gas flow velocity and interfacial tension, the behavior of the relative permeability curves in the presented model of this study shows good quantitative agreement with the reported experimental results by Jamiolahmady et al.^13^. With increasing flow rate due to the positive coupling effect, the amount of open throats in the network and consequently the relative permeability increase. According to the experimental results and the proposed model, this change is not remarkable for gas condensate. Enhancement of the surface tension has a negative effect on the relative permeability dependence. In fact, the amount of capillary force in the network is enhanced by increasing the surface tension and in turn reduces the number of open throats. In other words, by increasing the surface tension, the behavior of fluid flow is changed to a conventional two-phase flow phenomenon and each fluid move in a separate path in the network.

Using the results obtained in this investigation, the following conclusions can be stated:

- Implementing relative permeability curves of gas condensate in reservoir simulation software could result in optimum gas condensate production.
- Dynamic changes due to the relative permeability of gas and condensate as a function of saturation can be entered into the reservoir simulation to optimize inertia and positive coupling phenomena to maximized condensate production in gas condensate reservoir.
- The proposed ANN-based proxy model can promote the speed of calculation in gas condensate simulation, considering dynamic change of relative permeability curves as a function of gas condensate saturation.
- Application of proxy model, will results a fast computational protocol and using this advantage optimized adjustable coefficients of relative permeability curves of the gas condensate, which is recommended in professional software such as Eclipse with high accuracy.
- By entering the fractures pattern in the network model and predicting relative permeability of gas and condensate by suggested method the role of fractures in the production of gas condensate in such reservoirs will be predicted.

## Data Availability

The datasets used during the current study available from the corresponding author on reasonable request.
